# Proliferation of Cultured Mouse Choroid Plexus Epithelial Cells

**DOI:** 10.1371/journal.pone.0121738

**Published:** 2015-03-27

**Authors:** Basam Z. Barkho, Edwin S. Monuki

**Affiliations:** 1 Department of Pathology and Laboratory Medicine, University of California Irvine School of Medicine, Irvine, CA 92697, United States of America; 2 Sue and Bill Gross Stem Cell Research Center, University of California Irvine, Irvine, CA, 92697, United States of America; 3 Department of Developmental and Cell Biology, University of California Irvine School of Biological Sciences, Irvine, CA 92697, United States of America

## Abstract

The choroid plexus (ChP) epithelium is a multifunctional tissue found in the ventricles of the brain. The major function of the ChP epithelium is to produce cerebrospinal fluid (CSF) that bathes and nourishes the central nervous system (CNS). In addition to the CSF, ChP epithelial cells (CPECs) produce and secrete numerous neurotrophic factors that support brain homeostasis, such as adult hippocampal neurogenesis. Accordingly, damage and dysfunction to CPECs are thought to accelerate and intensify multiple disease phenotypes, and CPEC regeneration would represent a potential therapeutic approach for these diseases. However, previous reports suggest that CPECs rarely divide, although this has not been extensively studied in response to extrinsic factors. Utilizing a cell-cycle reporter mouse line and live cell imaging, we identified scratch injury and the growth factors insulin-like growth factor 1 (IGF-1) and epidermal growth factor (EGF) as extrinsic cues that promote increased CPEC expansion in vitro. Furthermore, we found that IGF-1 and EGF treatment enhances scratch injury-induced proliferation. Finally, we established whole tissue explant cultures and observed that IGF-1 and EGF promote CPEC division within the intact ChP epithelium. We conclude that although CPECs normally have a slow turnover rate, they expand in response to external stimuli such as injury and/or growth factors, which provides a potential avenue for enhancing ChP function after brain injury or neurodegeneration.

## Introduction

The choroid plexus (ChP), which resides in all four ventricles of the brain, produces and secretes cerebrospinal fluid (CSF). The major function of the CSF is to protect, nourish, and maintain homeostasis of the central nervous system (CNS) [1, 2]. Among their many beneficial functions, ChP epithelial cells (CPECs) are the main CNS source of transthyretin (TTR) [3]. This carrier protein transports thyroid hormone in the CSF and brain, and has been demonstrated to be a contributing factor to normal hippocampal neurogenesis [4, 5]. As well as their secretion function, CPECs form tight junctions that constitute the blood-CSF barrier [1, 6]. In injured and aging brains, CPEC pathologies—which include cell atrophy, barrier defects and reduced CSF and TTR production—are thought to be associated with disrupted brain homeostasis [7, 8]. Furthermore, these defects are accelerated in multiple brain disorders, such as Alzheimer disease, Amyotrophic lateral sclerosis, Huntington disease, Schizophrenia and Parkinson disease, and these CPEC defects are thought to intensify these CNS disorders (reviewed in [9]). Therefore, CPEC-based therapies could have applications in a variety of CNS dysfunctions and diseases.

Cell transplantation studies have suggested the therapeutic potential of CPECs for brain injury and disease [10, 11]. For example, transplanted ChP cells have a neuroprotective effect in rodent [12, 13] and monkey [14] neurodegeneration models. Recently, our lab derived human and mouse CPECs from embryonic stem (ES) cells, and demonstrated their capability to integrate into host mouse ChP epithelium [15]. However, consistent with cultured primary CPECs in vitro [16, 17], limitations exist to expanding ES cell-derived CPECs. Differentiation of neuroepithelial precursor cells into postmitotic CPECs occurs at early embryonic stages between embryonic day (E)11 and E18 [18, 19], and postnatal and adult CPECs display little to no proliferation or turnover in rodents [20], primates and humans [21, 22]. Correspondingly, CPECs have been difficult to expand in culture, which has limited the attempts to use CPECs for intraventricular injections, transplants, and other interventions. However, inducing CPEC proliferation has not been well investigated, and it remains unclear whether CPECs have the ability to divide in response to extrinsic stimuli, such as injury and growth factor treatment.

Using multiple cell proliferation assays, we demonstrate the cell division capacity of primary mouse CPECs in response to injury (scratch assay) and growth factor treatment (IGF-1 and EGF). We found that IGF-1 and EGF promote increased CPEC division when applied in combination, and enhance scratch-induced proliferation. Furthermore, in intact ChP tissue explant cultures, we observed CPECs entering the cell cycle in response to IGF-1 and EGF. Altogether, we provide some of the first evidence that extrinsic cues can promote the proliferation of postnatal mouse CPECs. The discovery of CPEC proliferative responses to extrinsic cues may have future applications for CPEC-based therapies in CNS diseases.

## Material and Methods

### Choroid Plexus Epithelial Cell (CPEC) dissection and culture

Isolation and culture of primary mouse CPECs was performed using modified methods previously established [23]. Primary cultures of enriched CPECs were prepared between postnatal day 4 to 10 (P4-P10) wild-type mouse pups (CD1 mice, Charles River Laboratories, Wilmington, MA), double transgenic Fucci (fluorescence ubiquitination-based cell cycle indicator) mice [24] and Ttr::RFP (Ttr promoter driving monomeric red fluorescent protein expression) transgenic mice [25]. All procedures involving live mice were performed according to approved Institutional Animal Care and Use Committee protocols and guidelines at University of California, Irvine. ChP tissue was isolated from the lateral, third and fourth ventricles, then pooled and treated with type II collagenase (Gibco, #17101) and TrypLE Express (Gibco, #12605–010) from Life Technologies (Carlsbad, CA) for 20 minutes each. For monolayer culture, cells were plated on poly-d-lysine/laminin coated plates at 6,500–13,000 cells/cm^2^ (unless stated otherwise) in DMEM with 10% FBS and 1X pen-strep solution (Gibco), and allowed to reach plate confluence (typically by 2–3 days). To eliminate mitotic cells which may include endothelial and stromal cells of the ChP, cytosine arabinoside (Ara-C) at 20 μM (Sigma-Aldrich; St. Louis, MO) was applied for 2–3 days [12, 26], and then cells were washed and allowed to recover for 24 hours before using in experiments. Based on morphology and immunocytochemistry, this generated a CPEC culture of approximately 90–95% purity. Culture media was changed every 2–3 days, and cells were maintained in a humidified 37°C incubator containing 5% CO_2_.

### Reagents

Primary rabbit anti-Cldn1 at 1:1000 (Invitrogen #71–7800) was used for staining. Following previously tested concentrations [27], recombinant IGF-1 (R&D Systems, #791-MG-050; Minneapolis, MN) was used at 100ng/mL, and recombinant EGF (Corning #CB40052; Corning, NY) was used at 20ng/mL, for 48 hours.

### Cell proliferation assay

Briefly, for cultured cells, 5-ethynyl-2′-deoxyuridine (EdU) was added to plated primary mouse CPECs at 10 μM final concentration for 48 hours, followed by fixation using 4% PFA. For in vivo proliferation analysis, adult mice were injected i.p. with 50 mg/g EdU, then sacrificed and perfused with 4% PFA after 24 hours. The Click-iT EdU cell imaging assay (#C10339; Molecular Probes) was used according to the manufacturer's instructions. Cultured cells or brain sections were washed with 1X PBS and blocked for 30 minutes, followed by incubation of the Click-iT cocktail reaction for an additional 30 minutes. For further cell marker staining, cells or tissue were washed with 1X PBS and processed for immunohistochemistry (see below).

### Immunocytochemistry, Microscopy and Quantification

Animals were perfused or cells were fixed using 4% PFA, followed by immunocytochemical staining and quantification. Unless stated otherwise, all images were obtained using a Zeiss LSM510 confocal microscope (Thornwood, New York). Z-stacks were obtained at 1 μm resolution. Results were statistically analyzed using 2-tailed, unpaired Student´s t-test, unless stated otherwise.

### Live-cell imaging

CPECs were placed on a glass bottom dish (MatTek Corp; Ashland, MA) as described above, and then imaged using an inverted confocal microscope outfitted with a thermo/CO2-regulated chamber and a computer-controlled motorized stage (Olympus FluoView FV10i; Tokyo, Japan). Bright field and/or fluorescent images (excitation at 470nm for mAG1 and 520nm for mKO) were obtained at 15- or 20-minute intervals with low exposure times and laser power (<10%) to minimize photo-bleaching, then edited into videos (FluoView Viewer software). For immunohistochemistry, cells were fixed and stained as described above, and imaged with the Nikon Eclipse Ti inverted microscope.

### Scratch assay

After establishing primary mouse CPEC confluency in a 6 well plate (see above), the monolayers were scratched with a 2 mm microspatula and immediately washed twice with 1X PBS before adding fresh media (modified from [28]). Live-cell images were obtained using the Olympus FluoView FV10i at 37°C and 5% CO_2_ with a 10X objective.

### Whole tissue explant cultures

After isolation, whole lateral, third and fourth ventricular ChP tissue were plated on Whatman nucleopore membranes (8 μm pores; Clifton, NJ) floating on media with Dispase (2mg/mL, Gibco #17105) at 37°C for O/N. After washing out the dispase and allowing tissue to settle for 24 hours, live-cell analysis was performed as described above. For EdU detection, we followed the manufacturer’s protocol (Click-iT Plus EdU kit; Molecular Probes #C10637).

## Results

### Injury increases CPEC proliferation in vitro

**Fig 1:**
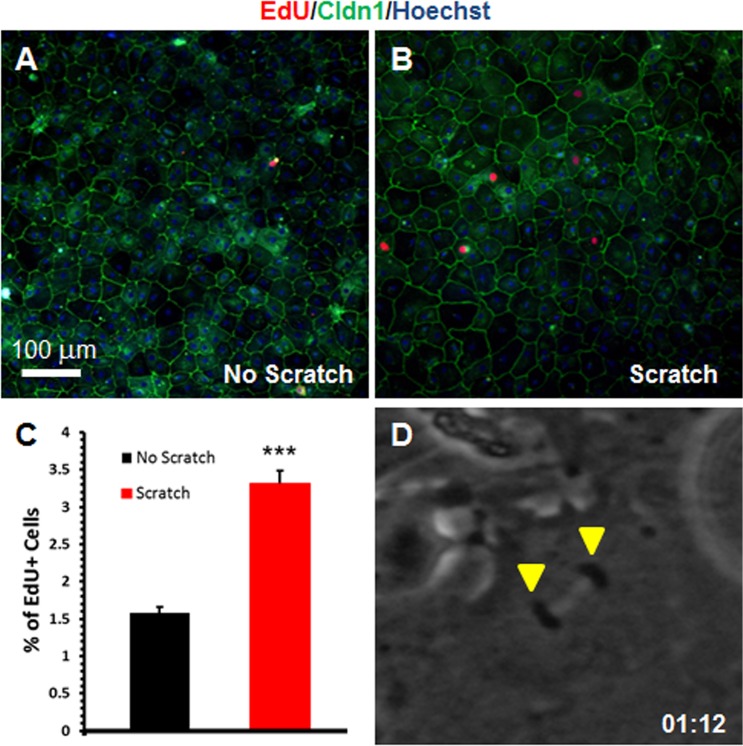
CPECs proliferate in response to scratch-wound injury. **(A-B)** Cell proliferation assay (EdU incorporation) with (B) and without (A) administering a scratch to a monolayer of mouse primary CPECs stained with Claudin 1 (Cldn1, green) and pulsed with EdU (red) for 48 hours. Nuclear stain = Hoechst (blue). Scale bar = 100 μm. **(C)** Percentage of EdU^+^ CPECs quantified from (A-B). Values represent mean±s.e.m. (n = 3 biological replicates each; ***P<0.001, Student´s t-test). **(D)** High magnification phase image of a CPEC in mitosis (anaphase stage) after a scratch (at 1 hr and 12 min from S1 Video, demonstrated by two arrowheads). Arrowheads designate the chromosomes and nuclei following sister chromatid separation.

To determine CPEC proliferation response to injury, we performed a wound assay on cultured primary mouse CPECs. First, to generate homogenous CPEC monolayer cultures and eliminate endothelial and stromal cell populations from the ChP tissue, we treated all four dissected ChP ventricles with cytosine arabinoside (Ara-C) [12]. As seen in Fig 1, this resulted in cultures enriched for CPECs based on classical "cobblestone" epithelial morphology and expression of the epithelial cell-specific tight junction marker Claudin 1 (Cldn1). After administering a manual scratch to these Ara-C-treated cultures, we detected higher EdU incorporation after 48 hours in Cldn1^+^ cells compared to unscratched cultures (Fig 1A and 1B). Cell counts revealed low baseline EdU incorporation in unscratched cultures, which increased two-fold in scratched regions (Fig 1C). Next, we monitored the primary CPEC cultures with live-cell imaging to observe cell invasion/spreading and mitotic events. We found that the scratch region was filled by adherent CPECs after 18 hours, and detected cell divisions in the region around 36 hours after the scratch (Fig 1D and S1 Video).

**Fig 2:**
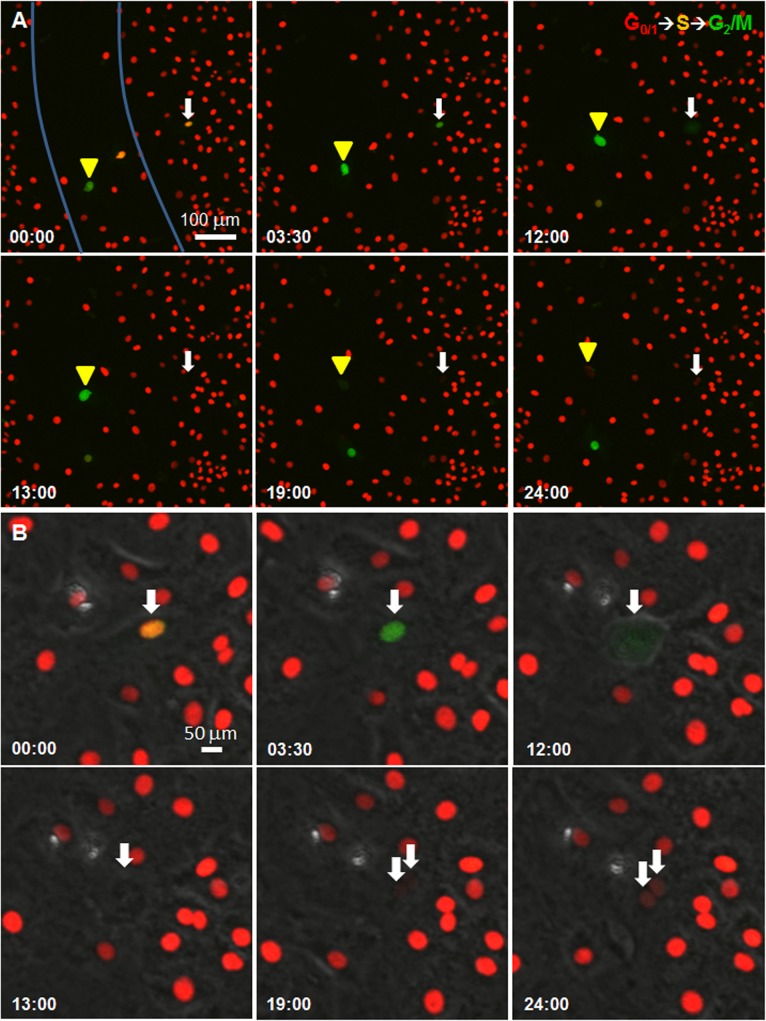
Scratch-wound injury increases CPEC proliferation. Sequential images (from S2 Video) of scratch assay on primary CPEC from Fucci mice. **(A)** White arrow (adjacent to scratch) and yellow arrowhead (within the scratch) show the nuclei of two cells in either S phase (mAG1 and mKO, orange nuclei) or G_2_/M phase (mAG1, green nuclei). By 24 hours, the nuclei have divided (mKO, paired red nuclei). Blue lines indicate scratch borders. Scale bar = 100 μm. **(B)** Magnified images (from S3 Video) of the cell designated by white arrow in (A), merged with phage contrast images. Scale bar = 50 μm.

To further assess CPEC proliferation within and around the scratch region, we utilized primary mouse CPEC monolayers derived from double transgenic fluorescent ubiquitination-based cell-cycle indicator (Fucci) mice. Cells from these mice express both monomeric Kusabira-Orange 2 (mKO2, at G_0_/G_1_) and Azami-Green 1 (mAG1, during S, G_2_ and M phase) [24]. In scratched cultures, we observed green mAG1 expression within and adjacent to the scratched region (Fig 2A and S2 Video). At higher magnification, we observed cell cycle progression from G_0_/G_1_ to S phase (onset of mAG1 expression) within 12 hours and completion of cell division (mKO2 expression in two daughter cells) within 24 hours (Fig 2B and S3 Video). Finally, to confirm that the invading/spreading and dividing populations are CPECs, we performed immunocytochemistry on the scratch samples and found that the majority of cells in the scratch region were Cldn1^+^. Furthermore, in regions far from or with no scratch, the vast majority of Cldn1^+^ cells expressed red mKO2 (S1A Fig), whereas mAG1 was expressed in Cldn1^+^ cells adjacent to the scratch (S1B Fig). Overall, the various cell proliferation methods provided clear evidence of increased primary CPEC proliferation in response to scratch injury.

### Simultaneous IGF-1 and EGF stimulation promotes increased CPEC proliferation

**Fig 3:**
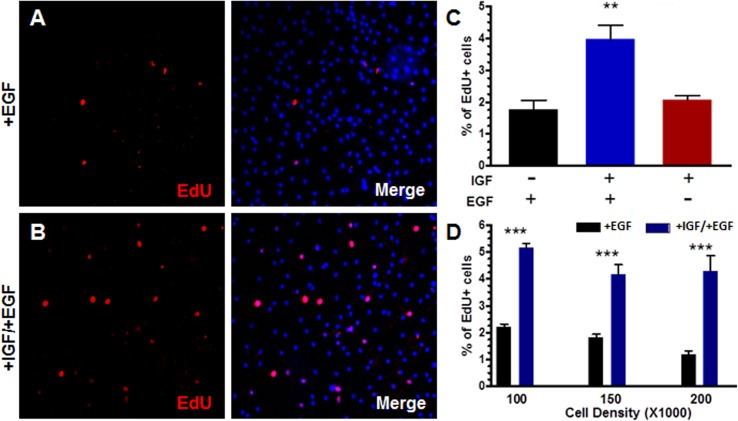
IGF-1 and EGF stimulation drives CPEC proliferation. **(A-B)** Mouse primary CPECs plated at 150,000 cell/well were treated with EGF alone (A) or IGF-1 and EGF together (B) and pulsed with EdU (red) for 48 hours. Right panels merged with nuclear Hoechst counterstain (blue). **(C)** Quantification of (A-B) showing percentage of EdU^+^ CPECs treated with IGF-1 and/or EGF. Values represent mean±s.e.m. (n = 3; **P<0.01, Student´s t-test). **(D)** Percentage of EdU^+^ CPECs plated at different cell densities with EGF +/- IGF-1. Values represent mean±s.e.m. (n = 3; black vs. blue bars—***P<0.001, Student´s t-test; no significance between black bars).

Insulin-like growth factor 1 (IGF-1) and epidermal growth factor (EGF) activate signaling cascades that regulate cell proliferation [29], and it is well known that IGF-1 and EGF signaling can stimulate the proliferation of epithelial cells in a range of tissues [29, 30]. Therefore, we evaluated whether IGF-1/EGF also induces CPEC division in vitro. First, we examined proliferation in response to varying combinations of the growth factors by EdU assay. Consistent with previous studies [27], we found that IGF-1 or EGF alone did not increase EdU incorporation significantly by 48 hours (compare Figs 1C and 3C). However, co-administration of IGF-1 and EGF caused a two-fold increase in EdU incorporation compared to either growth factor alone (Fig 3C). This effect was apparent at different cell densities spanning the 100-200K/well range (for a 6-well plate; Fig 3D). Overall, these findings demonstrate the ability of IGF-1 and EGF to stimulate primary CPEC proliferation.

### IGF-1 and EGF enhance injury-induced CPEC proliferation in monolayer and intact tissue cultures

**Fig 4:**
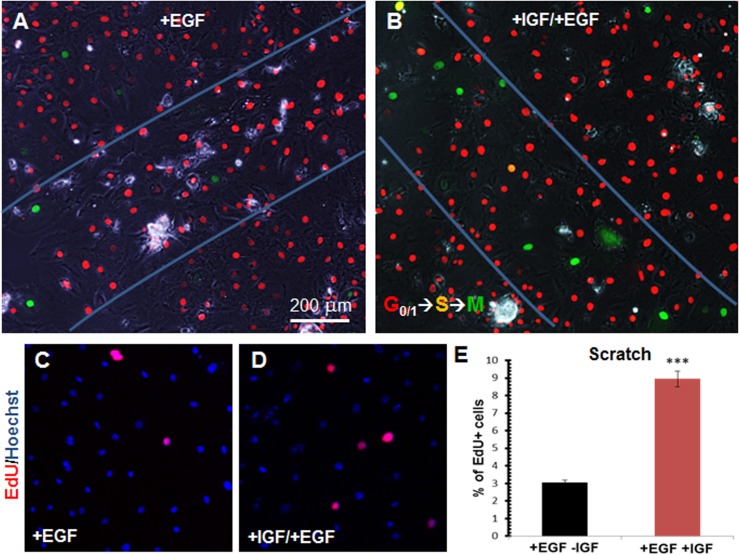
IGF-1 and EGF enhance CPEC injury-induced proliferation. **(A-B)** Scratch assay performed on cultured CPECs from Fucci mice and exposed to EGF alone (A) or both EGF and IGF-1 (B) over 22 hours post-scratch (from S4 and S5 Videos, respectively) with bright field image overlaid. Blue lines indicate scratch borders. Scale bar = 200 μm. **(C-D)** Wound assay of mouse CPECs pulsed immediately with EdU after administering a scratch, and treated with either EGF alone (C) or both IGF-1 and EGF (D). Images merged with nuclear Hoechst counterstain (blue). **(E)** Percentage of EdU^+^ CPECs treated with IGF-1 and EGF (C) compared to EGF alone (D) after administering a scratch. Values represent mean±s.e.m. (n = 3; ***P<0.001, Student´s t-test).

We then combined the growth factors and scratch injury, first utilizing the Fucci reporter cells. Within 24 hours of scratch injury to Fucci CPEC cultures (S4 and S5 Videos), we observed far more cells with induced expression of green mAG1 in IGF-1/EGF-treated cultures (Fig 4B) compared to EGF alone (Fig 4A). This effect was verified by EdU assay (Fig 4C and 4D), which revealed a three-fold increase in 48-hour EdU incorporation with both growth factors compared to EGF alone, and 9% EdU incorporation overall (Fig 4E). These findings demonstrate the ability of IGF-1 and EGF to further enhance the CPEC proliferation induced by scratch injury.

**Fig 5:**
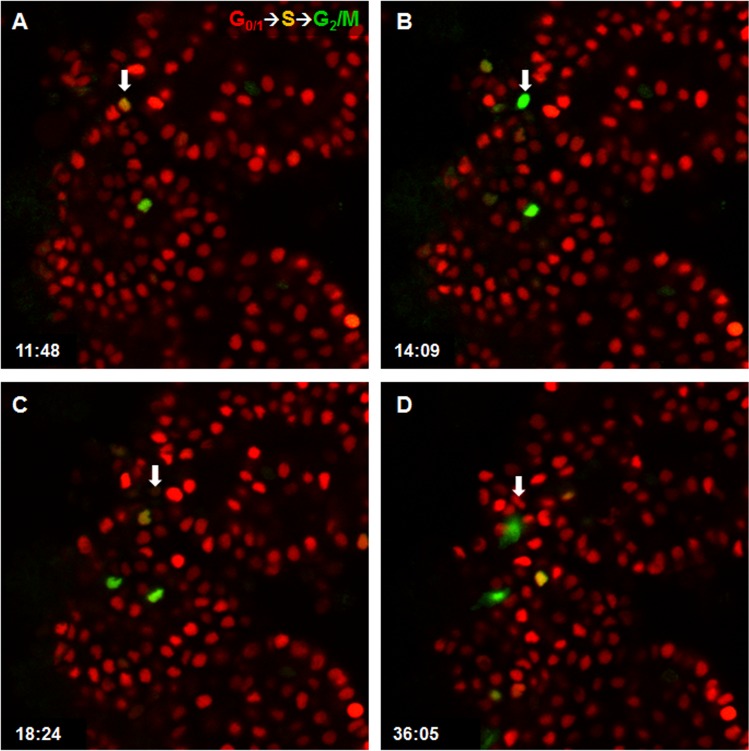
IGF-1 and EGF promotes CPEC proliferation in whole ChP explant cultures. **(A-D)** Sequential images of intact ChP tissue derived from Fucci mice treated with IGF-1 and EGF (from S6 Video). White arrow identifies the same cell across panels. S phase (mAG1 and mKO, orange nuclei) is detected at ~12 hours (A) and G_2_/M phases (mAG1, green nuclei) at ~14 hours (B). mAG1 expression is lost after 18 hours (C). After 32 hours, two cells are detected (mKO, two red nuclei) (D).

**Fig 6:**
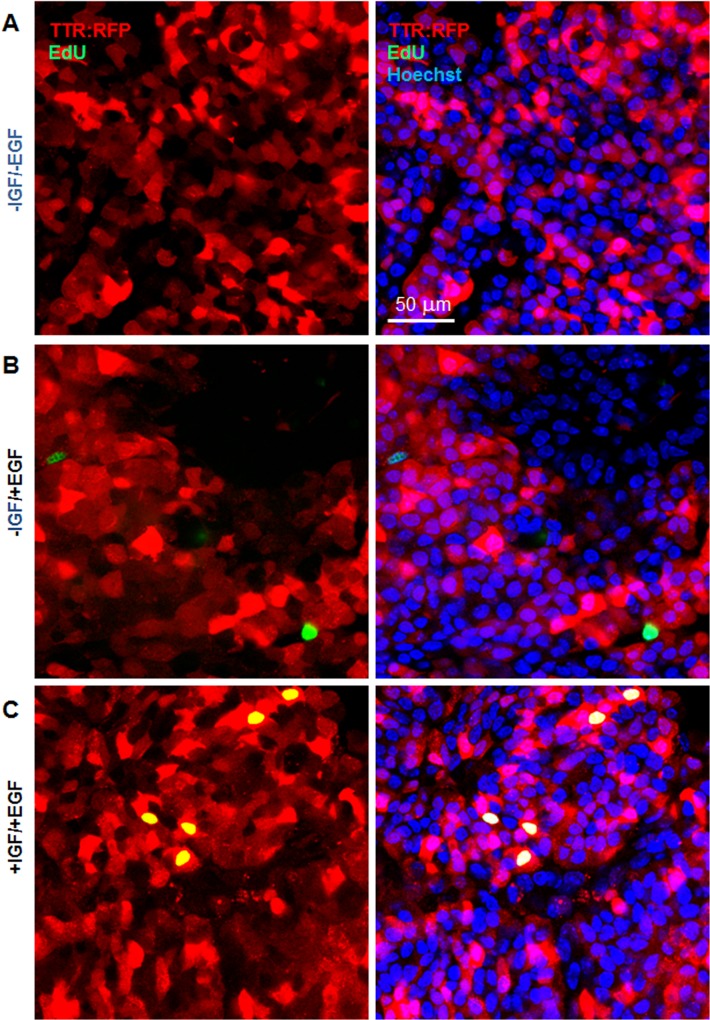
IGF-1 and EGF treatment increases CPEC proliferation in whole ChP explant cultures. **(A-C)** Intact ChP derived from Ttr::RFP mice treated with or without growth factors and pulsed with EdU (green) for 48 hours. Conditions include control, no growth factors (A), EGF alone (B) IGF-1 and EGF (C). Right panels merged with nuclear Hoechst counterstain (blue). EdU+ cells with no RFP expression could include stromal or endothelial cells of the ChP. Scale bar = 50 μm.

Next, to examine CPEC proliferation in intact ChP, we developed a whole ChP explant protocol (see Methods), and then analyzed time-lapse images of ChP explants from Fucci mice (S6 Video). In the explants, ChP cells entered S phase (co-expression of mAG1 and mKO2) around 12 hours after administering IGF-1 and EGF (Fig 5A). We then observed cells entering G_2_/M phase (Fig 5B) and the return of mKO2 expression in daughter cells around 32 hours (Fig 5D). In no growth factor or EGF alone conditions (S7 and S8 Videos, respectively), we observed little to no ChP cell proliferation. Next, to examine CPECs more specifically, we administered EdU to whole ChP explants from TTR::RFP mice [25], which express red fluorescent protein (RFP) in CPECs specifically [25]. Within 48 hours, we found that IGF-1 and EGF together increased EdU incorporation within RFP^+^ cells (Fig 6C) compared to no growth factor or EGF-only controls (Fig 6A and 6B). In conclusion, we found that IGF-1 and EGF stimulation promotes CPEC division within intact ChP epithelium.

## Discussion

Previous studies have reported a slow turnover rate for CPECs, and it remained unclear whether CPECs could proliferate in response to extrinsic cues. In this study, we provide evidence for extrinsically-enhanced CPEC proliferation in vitro. Our data from live-cell imaging of Fucci CPECs and ChP explants, as well as end-point EdU assays, provide clear evidence of increased CPEC division in response to different extrinsic cues—physical scratch injury and the growth factors, IGF-1 and EGF. IGF-1 and EGF together consistently yielded greater proliferation than either factor alone (Figs 3 and 6), and CPEC proliferation was most dramatic when IGF-1/EGF was combined with scratch injury (Fig 4). These studies demonstrate that primary mouse CPECs can be stimulated to proliferate in response to their extrinsic environment, and that the growth factor- and scratch-induced proliferation pathways may be independent.

The IGF-1 and EGF pathways are well known to affect cell proliferation, with crosstalk between these two pathways regulating the expression of key cell cycle regulators and cycle progression [27, 31]. For example, in other epithelial cells, EGF is thought to prime quiescent cells for G_0_-to-G_1_ transition, thereby allowing IGF-1 to promote progression into S phase [29]. This cooperative EGF/IGF-1 model would be consistent with our findings in primary CPECs, since CPEC division was not induced in the presence of either growth factor alone (Figs 3 and 6). It remains to be determined if the pathways downstream of IGF-1 and EGF, which have been previously implicated in epithelial proliferation (e.g. MEK, ERK, and PI3K-Akt pathways), play a role in CPEC proliferation, and whether autocrine/paracrine signaling plays a role in scratch-induced proliferation. Furthermore, IGF-1, -2 and EGF receptors are expressed by CPECs [32], supporting the possibility that subpopulations of CPECs exist with different receptor profiles.

CPEC proliferation in response to scratch injury, and its further enhancement by IGF-1/EGF, may have significance in human brain disorders. For example, in traumatic brain injury, multiple neurotrophic factors increase in the brain and CSF, including IGF-1 and EGF (reviewed in [5, 8], and these increases are thought to be due in part to increase ChP secretion. As described earlier, ChP secretion of neurotrophic factors is thought to be neuroprotective after brain injury and other CNS disorders [33, 34]. Furthermore, these disorders are also associated with ChP defects, which would lead to reduced secretion of neurotrophic factors. Proliferation within the ChP has been reported after rodent ischemic stroke [35], suggesting ischemic injury as a potential extrinsic CPEC mitogen, although additional experiments will be required to study CPEC proliferation specifically and whether the extrinsic factors defined in our study can facilitate CPEC proliferative responses in vivo.

While we observed CPEC proliferation to extrinsic cues, induced proliferation was somewhat modest overall. The largest effect was seen when scratch injury was combined with IGF-1/EGF (9% EdU incorporation by 48 hours), while the individual factors resulted in lower degrees of induced proliferation. This would suggest a relatively strong intrinsic constraint on CPEC cycle re-entry and division. This would be consistent with the low levels of in vivo CPEC turnover described previously, and maximal CPEC cycle re-entry and progression may require direct alterations of cell cycle inhibitors, such as the Cip/Kip (CDK interacting protein/Kinase inhibitory protein) and INK4a (Inhibitor of Kinase 4) proteins. Further studies on normal, injured, and degenerated ChP may identify the extrinsic factors and intrinsic cell cycle regulators of potential therapeutic relevance, and further enhance the regenerative potential of CPECs in brain damage or neurodegenerative diseases.

## Supporting Information

S1 Fig (A) Immunohistochemistry of primary mouse CPECs monolayer culture with no scratch for Cldn 1 (cell surface) with co-localization of mKO expression (red, nuclei) and no mAG1 expression (green, nuclei) after 48 hours. **(B)** Immunohistochemistry of CPECs (Cldn 1) 48 hrs after a scratch was administered with co-localization of mAG1 (green, nuclei). Right panels merged with nuclear Hoechst counterstain (blue).
(TIF)

S1 Video CPEC spreading/invasion and division following a scratch. (MP4)

S2 Video Fucci-derived CPECs scratch assay. (MP4)

S3 Video Fucci-derived CPECs scratch assay with bright field. (MP4)

S4 Video Fucci-derived CPECs scratch assay with no growth factors treatment. (MP4)

S5 Video Fucci-derived CPECs scratch assay with IGF-1 and EGF treatment. (MP4)

S6 Video Fucci-derived ChP tissue explants with IGF-1 and EGF treatment. (MP4)

S7 Video Fucci-derived ChP tissue explant with no growth factor treatment. (MP4)

S8 Video Fucci-derived ChP tissue explant with EGF treatment. (MP4)
