# Supplementary figures and images for: Proliferation of Cultured Mouse Choroid Plexus Epithelial Cells
Source: PLoS One. 2015 Mar 27;10(3):e0121738. doi: 10.1371/journal.pone.0121738 (PMC4376882; doi:10.1371/journal.pone.0121738)

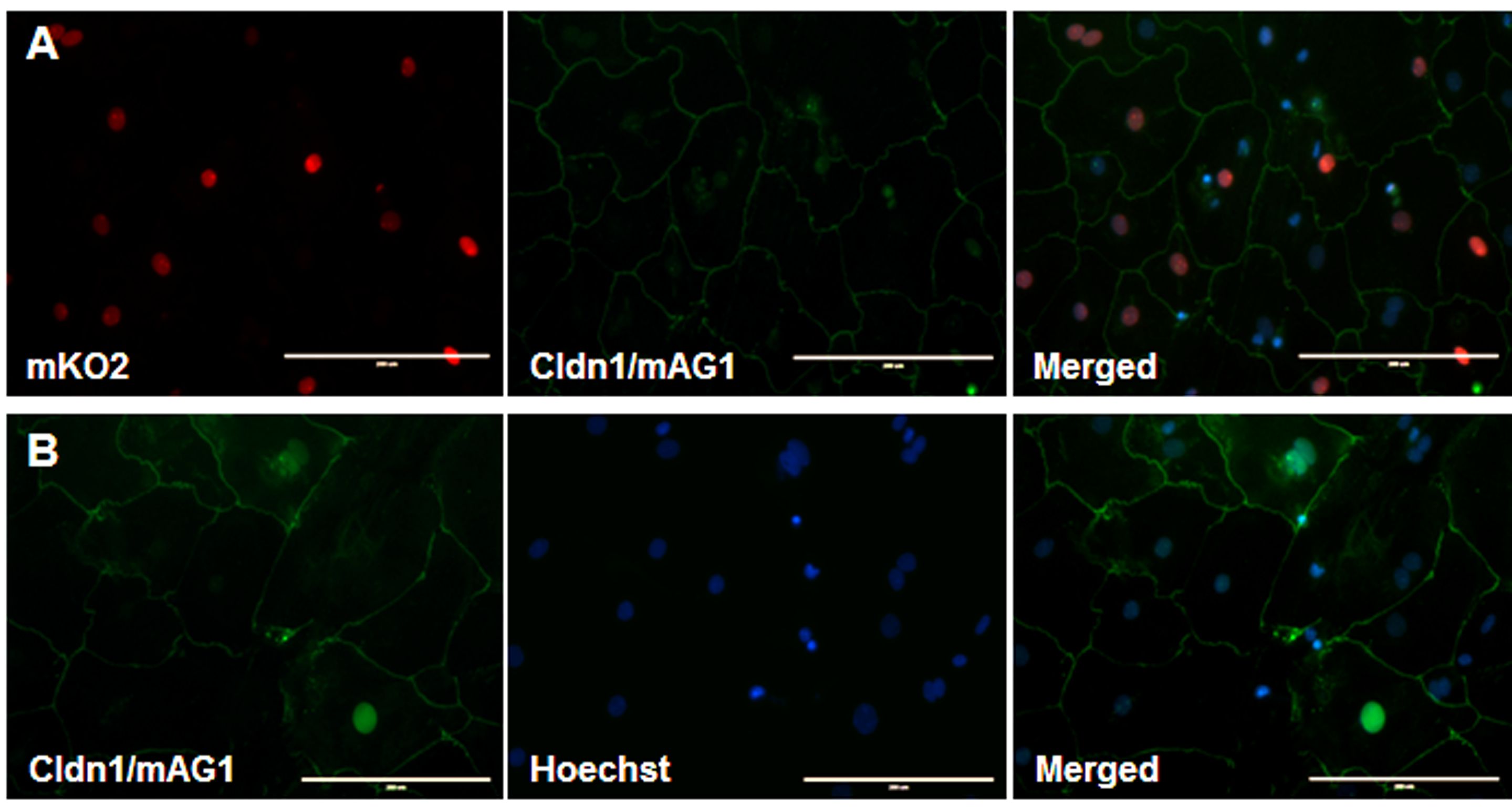

Supplement: S1 Fig — (B) Immunohistochemistry of CPECs (Cldn 1) 48 hrs after a scratch was administered with co-localization of mAG1 (green, nuclei). Right panels merged with nuclear Hoechst counterstain (blue). (TIF) [file pone.0121738.s001.tif]
